# High-Fat Diet-Induced Trefoil Factor Family Member 2 (TFF2) to Counteract the Immune-Mediated Damage in Mice

**DOI:** 10.3390/ani11020258

**Published:** 2021-01-21

**Authors:** Abdelaziz Ghanemi, Mayumi Yoshioka, Jonny St-Amand

**Affiliations:** 1 Department of Molecular Medicine, Faculty of Medicine, Laval University, Québec, QC G1V 0A6, Canada; abdelaziz.ghanemi@crchudequebec.ulaval.ca; 2 Functional Genomics Laboratory, Endocrinology and Nephrology Axis, CHU de Québec-Université Laval Research Center, Québec, QC G1V 4G2, Canada; mayumi.yoshioka@crchudequebec.ulaval.ca

**Keywords:** trefoil factor family member 2 (TFF2), high-fat diet, immunity, damage, mice

## Abstract

**Simple Summary:**

High-fat (HF) diet induces both immune-mediated damage and trefoil factor family member 2 (*Tff2*) expression. As TFF2 has tissue repair and protection properties, this suggests that HF diet-induced *Tff2* production and the resulting TFF2 mucosal protective effects would be a mechanism to counteract the HF diet-induced tissue damage. On the other hand, the induction of *Tff2* by HF diet could indicate that TFF2 is a food intake regulator (appetite control) since *Tff2* is also expressed in the brain. This highlights the importance of exploring TFF2-related pathways in the context of obesity management towards potential therapies.

**Abstract:**

Physiological homeostasis requires a balance between the immunological functions and the resulting damage/side effects of the immunological reactions including those related to high-fat (HF) diet. Within this context, whereas HF diet, through diverse mechanisms (such as inflammation), leads to immune-mediated damage, trefoil factor family member 2 (*Tff2*) represents a HF diet-induced gene. On the other hand, TFF2 both promotes tissue repair and reduces inflammation. These properties are towards counteracting the immune-mediated damage resulting from the HF diet. These observations suggest that the HF diet-induction of *Tff2* could be a regulatory pathway aiming to counteract the immune-mediated damage resulting from the HF diet. Interestingly, since *Tff2* expression increases with HF diet and with *Tff2* also expressed in the brain, we also hypothesize that TFF2 could be a HF diet-induced food intake-control signal that reduces appetite. This hypothesis fits with counteracting the immune damage since reducing the food intake will reduce the HF intake and therefore, reduces the HF diet-induced tissue damage. Such food intake signaling would be an indirect mechanism by which TFF2 promotes tissue repair as well as a pathway worth exploring for potential obesity management pharmacotherapies.

Animal physiological homeostasis requires a balance between the immunological functions and the damage/side effects of those immunological reactions. Knowing that immunological reactions can be triggered by diverse factors, the homeostasis supposes that parallel or secondary pathways are activated or stimulated with these immunological reactions to repair the damage. The immune system is a complex network of cells and circulating fluids that is modulated by the nervous system [1], endocrine system [2], infections [3], and even diet. Indeed, different types of diets, such as high-sucrose and high-fat (HF) diets, have been shown to impact immune functions [4,5], among other factors and genes [6,7]. HF diets characterize our modern life, and are associated with diverse diseases and health problems, such as obesity, dyslipidemia, diabetes, fatty liver disease and cardiovascular diseases [7,8,9,10]. However, such HF diet-induced immune modulations, which could be implicated in the HF diet-induced risks and diseases, are yet to be fully understood. Within this context, the molecules and signals that are either upregulated or downregulated with HF diets could be the mechanistic answer, as per the examples we provide below from studies on mice. 

For instance, trefoil factor family member 2 (TFF2), known as spasmolytic peptide [11], is well involved in mucosal repair, protection and proliferation, as it represents an important stabilizer of the gastric mucus, with roles in tissue remodeling [12]. Herein, we go beyond its mucosal protective role to explore the hypothesis linking this diet-induced molecule, TFF2, to the diet-induced immunomodulation. Indeed, whereas *Tff2* has been reported as a gene that is specifically induced by HF diets in mice [13,14], its knockout protected mice from HF diet-induced obesity [15] through a metabolic phenotype that contributes to more energy expenditure and reduced energy storage [16]. The importance of the studies that identified *Tff2* as a gene specifically induced by HF diets is that the control groups were, unlike in other studies, fasted mice [13,14]. Based on the HF induction of TFF2, we notice a correlation between the HF diet-induced immunological changes and the TFF2-related immunological effects and benefits (as illustrated below). This correlation suggests that TFF2 would be involved in mediating the protective effects against such HF diet damage.

On one side, a HF diet has important immunological impacts. For instance, a HF diet increases TNFα and IL1β in young mice’s hippocampus [17], and leads to chronic systemic inflammation [18]. Moreover, a chronic HF diet is also associated with obesity [19,20], which also affects the immunity [21] and might explain some of the impacts obesity has on regeneration impairment through diverse processes, including inflammation [22], which is important in the context of TFF2′s roles in tissues repair. 

On the other hand, TFF2, beyond its well-known roles in injured mucosa healing [23,24,25], has a noticeable role in the immune response [25,26], as suggested by its expression in immune organs [27] and its expression during inflammations [12]. Indeed, *Helicobacter* infection upregulated it in gastric tissues, macrophages and lymphocytes [11], whereas *Helicobacter pylori* eradication decreased TFF2 level in patients’ sera [28]. Furthermore, TFF2 deficiency leads to a deregulation of macrophages’ and lymphocytes’ proliferative responses [11], and an accelerated gastritis progression [29] during *Helicobacter* infection. This correlates with both the ulceration role of *Helicobacter pylori* [30] and the tissue repair/protections roles of TFF2 in animal selected tissues [12].

TFF2 expression during such immunological changes seems to be an attempt to limit the negative impacts of these immune reactions, such as inflammation [12], due to the HF diet. For instance, TFF2 could both limit the recruitment of leukocytes and the monocyte production of nitric oxide [25], and decrease macrophage responsiveness [27], which would contribute to promoting the tissue repair environment. Therefore, this TFF2- induced downregulation of selected immunological responses would be a step required to accomplish the healing and protecting effects TFF2 governs.

These illustrative examples present TFF2 as a mediator of the HF diet-triggered mechanisms attempting to correct the HF diet’s negative impacts, mediated through the immune system. Interestingly, unlike glucose, which causes insulin as a hormone to be secreted immediately following meal ingestion [31], there is no equivalent hormone for lipid ingestion. TFF2 could be that missing signal within animal endocrinology, since in the studies in which *Tff2* was shown to be unregulated at 3 h following a low-fat meal ingestion, it was upregulated with a HF meal [13,14]. The acute character of this expression indicates an immediate effect of the HF diet on Tff2 expression. Therefore, TFF2 could be a short-term lipid-specific signal that controls lipid intake by limiting lipid ingestion through a TFF2-dependant feedback acting on food intake centers. This is supported by the differential *Tff2* expression in the hypothalamus of fasted, and low-fat and HF diet- fed, mice (lipid ratio-dependent expression) [15]. This hypothesis is further supported by the increase in the drive to consume a HF meal, as well as the appetite enhancement as a consequence of TFF2 deficiency [15]. This would suggest that TFF2 counteracts HF diet-induced damage indirectly through reducing the HF intake. The other remarkable link is that TFF2 is mostly expressed in the digestive system [32,33], which represents the site whereat the animal’s neuroendocrine receptors first interact with the ingested food, including HF meals; this further suggests the acute responsiveness of the HF diet’s induction of TFF2 in the mouse intestine. Always within the digestive system, the HF diet impacts the local microbiome [34,35], which could be another key link between the diet and the immunological changes, especially with the known interactions between the immune system and the microbiome [36,37,38], the microbiota richness reduction [39], and dysbiosis, in all of which the HF diet has been implicated [40]. In addition, since several effects of a HF diet are mediated by microbiota [18] with probiotics that upregulate TFF2 [41], these microbiota-mediated effects of the HF diet could be through TFF2 expression changes. 

These elements highlight TFF2 expression (HF diet-induced) as a feedback aiming to counteract the immune-mediated HF diet-induced damage. However, the correcting potential and efficacy of TFF2 would depend on the severity and the chronic or acute character of such a HF diet. This explains why during obesity (such as in HF diet-induced obesity in animal models), those TFF2-correcting mechanisms are less efficient due to the strong immune-mediated damage that overcomes the TFF2-counteracting ability. Further explorations of diets’ impacts on TFF2 expression, such as high-salt diets [42], within an immunological context would expand this emerging field linking the type of diet to the immunological changes via identifying the linking factors. Importantly, combining these metabolic and immunological properties of TFF2 would allow us to further understand how mice immunologically react to a HF diet, and elucidate more diet-induced effects on immunology, infections and inflammation. Importantly, extrapolating these concepts from mice to humans and building clinical trials based on animal experiments could lead to developing novel TFF2-based therapies for diseases and conditions, such as inflammation, and, most importantly, a potential control for lipid intake (appetite control) towards a better obesity management strategy, which requires urgent solutions due obesity’s epidemiological profile and its impacts on health and the economy [43,44,45,46].

## Data Availability

Not applicable.
